# The benign c.344G > A: p.(Arg115His) variant in the *LDLR* gene interpreted from a pedigree-based genetic analysis of familial hypercholesterolemia

**DOI:** 10.1186/s12944-020-01252-4

**Published:** 2020-04-06

**Authors:** Mika Hori, Atsushi Takahashi, Cheol Son, Masatsune Ogura, Mariko Harada-Shiba

**Affiliations:** 1 Department of Molecular Innovation in Lipidology, National Cerebral and Cardiovascular Center Research Institute, 6-1 Kishibe-Shimmachi, Suita, Osaka, 564-8565 Japan; 2 Department of Genomic Medicine, National Cerebral and Cardiovascular Center Research Institute, 6-1 Kishibe-Shimmachi, Suita, Osaka, 564-8565 Japan; 3 Laboratory of Clinical Genetics, National Cerebral and Cardiovascular Center, 6-1 Kishibe-Shimmachi, Suita, Osaka, 564-8565 Japan

**Keywords:** LDL receptor, Familial hypercholesterolemia, Variant, Benign, Annotation

## Abstract

**Background:**

We previously identified the c.344G > A: p.(Arg115His) variant in the low-density lipoprotein receptor (*LDLR)* gene, which was interpreted as “conflicting interpretations of pathogenicity” in ClinVar, based on a genetic analysis of patients with familial hypercholesterolemia (FH). However, whether this variant affects the pathophysiology of FH remains unclear. Therefore, our aim was to annotate the c.344G > A: p.(Arg115His) variant in the *LDLR* gene in FH. We present 2 families harboring the c.344G > A: p.(Arg115His) variant in the *LDLR* gene.

**Methods:**

Genetic analyses were performed for the coding regions and the exon-intron boundary sequence of the *LDLR* and proprotein convertase subtilisin/kexin type 9 (*PCSK9)* genes in 2 FH families. Next, the family without pathogenic variants in the *LDLR* and *PCSK9* genes was screened by whole-exome sequencing. Detailed clinical and biochemical data were gathered from family members.

**Results:**

In one family, the index case had biallelic c.1567G > A: p.(Val523Met) and c.344G > A: p.(Arg115His) variants in the *LDLR* gene, while the sibling had only the c.1567G > A: p.(Val523Met) variant in the *LDLR* gene. There was no difference in the FH phenotype between the siblings. In another family, the index case and the sibling had no pathogenic variants in the *LDLR*, *PCSK9*, and apolipoprotein B (*APOB)* genes, but the sibling’s wife with nonFH had the c.344G > A: p.(Arg115His) variant in the *LDLR* gene. The sibling and his wife had 4 children, including an unaffected child and an affected child who had the c.344G > A: p.(Arg115His) variant in the *LDLR* gene. In addition, the allele frequency of the c.344G > A: p.(Arg115His) variant (0.0023–0.0043) in Japanese and East Asian populations is relatively high compared with that of the other *LDLR* pathogenic variants (0.0001–0.0008).

**Conclusions:**

The c.344G > A: p.(Arg115His) variant in the *LDLR* gene is interpreted as benign in individuals with FH.

## Background

Familial hypercholesterolemia (FH) is a disease that leads to a high risk of coronary artery disease (CAD) because FH patients are exposed to high serum LDL-cholesterol (LDL-C) levels from birth. The prevalence of FH is 1 per 200–500 individuals in the general population [1]. FH is caused by mutations in the low-density lipoprotein receptor (*LDLR*) and in the related genes apolipoprotein B (*APOB*) and proprotein convertase subtilisin/kexin type 9 (*PCSK9*). More than 4970 variants in the *LDLR* gene, 580 variants in the *APOB* gene, and 350 variants in the *PCSK9* gene are shown in ClinVar [2]. In Japan, pathogenic variants in the *APOB* gene have not been reported [3]. Whether some variants of the *LDLR* and *PCSK9* genes affect the pathophysiology of FH remains unclear.

In our FH cohort, patients were found to harbor the c.344G > A: p.(Arg115His) variant in the *LDLR* gene, which was interpreted as “conflicting interpretations of pathogenicity” in ClinVar. However, whether this variant affects the pathophysiology of FH remains to be elucidated. Herein, we present 2 families harboring the c.344G > A: p.(Arg115His) variant in the *LDLR* gene, including an index case with biallelic *LDLR* variants that comprised the c.344G > A: p.(Arg115His) and a pathogenic variant (Family 1) and nonFH patients with the c.344G > A: p.(Arg115His) variant (Family 2).

## Methods

The protocol of this study was approved by the Ethics Review Committee of the National Cerebral and Cardiovascular Center (M17–56 or M24–80). Each patient provided written informed consent to participate in the study.

### DNA analysis

Genomic DNA was extracted from whole blood from the sibling of Family 1 and the index case of Family 2 using an automated DNA extraction machine (QIAsymphony; QIAGEN, Valencia, CA) and from the other members of Family 2 using the Genome Extraction Kit (GENOMIX; Biologica, Nagoya, JAPAN) by SRL Inc. (Tokyo, JAPAN). The coding regions and exon-intron boundary sequences of the *LDLR* and *PCSK9* genes were examined by Sanger sequencing as described previously [4]. For the index case of Family 2, multiplex ligation-dependent probe amplification (MLPA) was performed to detect large rearrangements of the *LDLR* gene using the P062B LDLR MLPA Kit (MRC Holland, Amsterdam, the Netherlands). Next, whole-exome sequencing was performed for Family 2. Exome libraries were prepared using the SureSelect Human All Exon V7 Kit (Agilent Technologies, Santa Clara, CA). Sequencing was performed by NovaSeq 6000 (Illumina, San Diego, CA) with 150 bp paired-end reads at RIKEN GENESIS CO., LTD. Sequence reads were aligned to the human reference genome (hg19) using BWA-MEM. Single nucleotide variants and small indels were called with HaplotypeCaller of the Genome Analysis Tool Kit. The presence/absence of the c.344G > A:p.(Arg115His) variant in the *LDLR* gene was confirmed by Sanger sequencing.

### Clinical and laboratory data

Serum total cholesterol (TC), triglycerides (TG), and high-density lipoprotein-cholesterol (HDL-C) levels were measured using enzymatic methods. LDL-C levels were calculated by the Friedewald formula or were measured using enzymatic methods. Achilles tendon thickness (ATT) was measured by X-ray. CAD was evaluated by the presence of myocardial infarction, angina pectoris, or coronary arteries with ≥ 75% stenosis by coronary angiography or electrocardiogram.

## Results

### Case presentation

#### Family 1

**Fig. 1:**
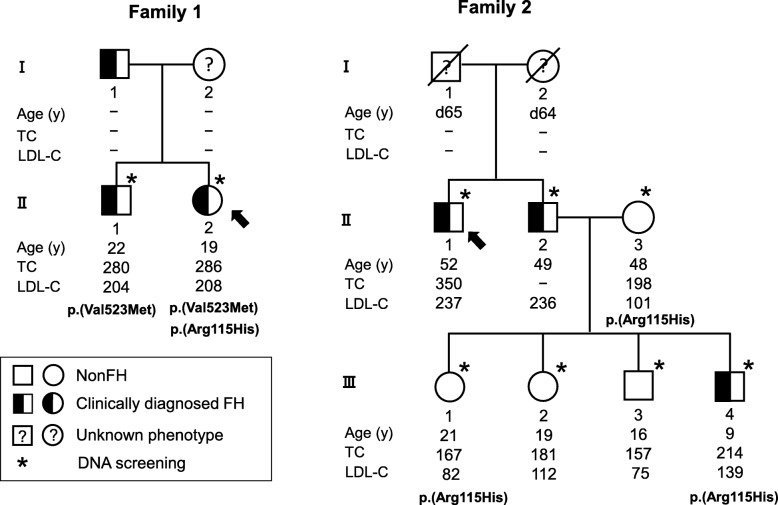
Two family pedigrees with the c.344G > A: p.(Arg115His) variant in the *LDLR* gene. Arrows show the index cases

The index case was a 19-year-old woman who had biallelic *LDLR* c.1567G > A: p.(Val523Met) and c.344G > A: p.(Arg115His) variants (Fig. 1, Table 1). She was referred to our lipid clinic with her brother at the age of 19 years for dyslipidemia, and her untreated LDL-C level was 208 mg/dL. She did not have ATT. Her medications included 2.5 mg of rosuvastatin, and her LDL-C level was 100 mg/dL on this medication regimen. Her sibling (II-1) was a 21-year-old male who had the *LDLR* c.1567G > A: p.(Val523Met) variant. His untreated LDL-C level was 204 mg/dL at 21 years old. His ATT values were 6.7 and 9.1 mm. His medication included 2.5 mg of rosuvastatin, and his LDL-C level was 99 mg/dL under this medication regimen. Their father was diagnosed with FH, but his lipid profile was unknown.

**Table 1:** Clinical and genetic characteristcs of the family with the c.344G > A: p.(Arg115His) variant in the *LDLR* gene

Family	1		2						
Case	II-1	II-2	II-1	II-2	II-3	III-1	III-2	III-3	III-4
Clinical diagnosis	FH	FH	FH	FH	nonFH	nonFH	nonFH	nonFH	FH
Age (y)	22	19	52	49	48	21	19	16	9
Sex	M	F	M	M	F	F	F	M	M
TC (mg/dL)	280	286	350	–	198	167	181	157	214
TG (mg/dL)	42	58	239	137	262	77	53	71	153
HDL-C (mg/dL)	68	66	–	49	45	70	58	68	44
LDL-C (mg/dL)	204	208	237	236	101	82	112	75	139
Achilless tendon thickness	Yes	No	Yes	Yes	No	No	No	No	No
Xanthoma	No	No	No	No	No	No	No	No	No
Corneal Acrus	No	No	Yes	Yes	No	No	No	No	No
CAD	No	No	No	No	No	No	No	No	No
*LDLR* pathogenic variant	c.1567G > A: p.(Val523Met)	c.1567G > A: p.(Val523Met)	None	None	None	None	None	None	None
*LDLR* variant	None	c.344G > A: p.(Arg115His)	None	None	c.344G > A: p.(Arg115His)	c.344G > A: p.(Arg115His)	None	None	c.344G > A: p.(Arg115His)
*PCSK9* pathogenic variants	None	None	None	None	None	None	None	None	None

*CAD* coronary artery disease

Untreated serum lipid levels are shown

#### Family 2

The index case was a 52-year-old man who had no pathogenic variants in the *LDLR* and *PCSK9* genes (Fig. 1). He was referred to the lipid clinic at the age of 52 years for dyslipidemia (II-1). His untreated LDL-C level was 237 mg/dL, and his ATT values were 7.1 mm and 9.2 mm. He was diagnosed with hypertension at the age of 45 years. His medication regimen included 5 mg of rosuvastatin and 10 mg of ezetimibe, and his LDL-C level was 111 mg/dL under this regimen. The lipid profiles of his parents were unknown. His father died of liver cancer at 65 years old, and his mother died at 64 years old due to unknown causes. The sibling (II-2) of the index case was a 49-year-old man who was diagnosed with FH. His medication regimen included 2 mg of rosuvastatin, 10 mg of ezetimibe, and 2 g of omega-3 fatty acid ethyl, and his LDL-C level was 106 mg/dL under this regimen. The index case and the sibling’s family were screened by whole-exome sequencing. We examined the coding regions and exon-intron boundary sequences in the *LDLR*, *PCSK9*, and *APOB* genes. The index case and the sibling had no pathogenic variants in the *LDLR, PCSK9,* and *APOB* genes, but the wife of the sibling (II-3) with nonFH had the c.344G > A: p.(Arg115His) variant in the *LDLR* gene. They had no loss-of-function mutations in the *PCSK9* and *APOB* genes. The sibling and the wife had 4 children, of whom 3 siblings (III-1, III-2, and III-3) were diagnosed with nonFH and 1 sibling (III-4) was diagnosed with FH. The eldest daughter (III-1) and the youngest son (III-4) were heterozygous for the c.344G > A: p.(Arg115His) variant in the *LDLR* gene. The presence/absence of the c.344G > A:p.(Arg115His) variant in the *LDLR* gene was confirmed by Sanger sequencing.

Allele frequency of the c.344G > A: p.(Arg115His) variant in the *LDLR* gene in Japanese and East Asian populations.

The allele frequency of the c.344G > A: p.(Arg115His) variant in the *LDLR* gene in Japanese and East Asian populations was 0.0029, 0.0043, and 0.0023 according to the Japanese Human Genetic Variation Database (*n* = 1201; HGVD: http://www.genome.med.kyoto-u.ac.jp/SnpDB) [5], the Tohoku Medical Megabank Organization database (*n* = 3381; tommo_3.5KJPN) [6], and ExAC (*n* = 66,000, [7]), respectively. The allele frequency of the c.344G > A: p.(Arg115His) variant in the *LDLR* gene was relatively high compared with that of the other *LDLR* pathogenic variants (0.0001–0.0008) detected in these databases.

## Discussion

We present 2 families harboring the c.344G > A: p.(Arg115His) variant in the *LDLR* gene. In the first family (Family 1), we identified an index case of biallelic *LDLR* variants that included c.1567G > A: p.(Val523Met) and c.344G > A: p.(Arg115His), and in the second family (Family 2), we identified that the wife of the index case’s sibling with FH, who was normolipidemic, had the *LDLR* c.344G > A: p.(Arg115His) variant. In Family 1, the index case had biallelic *LDLR* variants that included c.1567G > A: p.(Val523Met) and c.344G > A: p.(Arg115His), while the sibling had only the heterozygous c.1567G > A: p.(Val523Met) variant in the *LDLR* gene. The c.1567G > A: p.(Val523Met) variant is defined as pathogenic/likely pathogenic in ClinVar. FH patients harboring biallelic *LDLR* pathogenic variants generally show a much more severe phenotype than those harboring heterozygous *LDLR* pathogenic variants. However, the phenotype of the index case in Family 1 was identical to that of heterozygous FH. In Family 2, the index case’s sibling with FH had 4 children, namely, 1 affected and 3 unaffected siblings. His wife with nonFH had the c.344G > A: p.(Arg115His) variant in the *LDLR* gene. A heterozygous c.344G > A: p.(Arg115His) variant in the *LDLR* gene was detected in 1 unaffected sibling and 1 affected sibling. The FH phenotype of the youngest son is suggested to be derived from his father harboring unknown pathogenic variants.

The c.344G > A: p.(Arg115His) variant in the *LDLR* gene has been reported in several individuals of Asian ethnicity [3, 8]. The allele frequency of the c.344G > A: p.(Arg115His) (0.029/0.0043) variant in the *LDLR* gene among the Japanese population was similar to that expected based on the prevalence of heterozygous FH among the Japanese population (0.002–0.005). The allele frequency of the c.344G > A: p.(Arg115His) variant in the *LDLR* gene is relatively high compared with that of other *LDLR* pathogenic variants (0.0001–0.0008) in Japanese databases. The *LDLR* gene is located on chromosome 19p13.1–13.3 and contains 18 exons, encoding a mature protein of 839 amino acids with a 21 amino acid signal peptide. The c.344G > A: p.(Arg115His) variant is located in the cysteine-rich, 40 amino acid repeat region of the binding domain of the LDLR, but the 115th Arg is not conserved among this region [9]. In addition, the Arg (pI = 10.76) to His (pI = 7.59) substitution does not change the positive charge of this sequence. For the c.344G > A: p.(Arg115His) variant, only one study exists; functional assay results revealed that receptor activities were 64% of normal in COS7 cells transfected with *LDLR* cDNA containing the c.344G > A: p.(Arg115His) variant [10]. However, the genotype-phenotype correlation of the c.344G > A:p.(Arg115His) in the *LDLR* gene in pedigrees has not been demonstrated. Thus, based on 2 pedigree-based genetic analyses, the c.344G > A: p.(Arg115His) variant in the *LDLR* gene was classified as benign according to the guidelines issued by the American College of Medical Genetics and Genomics and the Association for Molecular Pathology. Thus, examining the genotype-phenotype correlation in a pedigree is important for providing a genetic diagnosis with high accuracy.

In our cohort, we showed that unrelated patients harboring no pathogenic variants in the *LDLR* and *PCSK9* genes comprised approximately 40% of FH patients [4]. FH may be partly explained by the accumulation of common SNPs [11]. A number of factors, including somatic genetic changes, environmental factors, and other genetic factors, might contribute to the pathogenesis and phenotypic variations observed in FH. We are currently searching for new candidate FH genes that may be responsible for FH in Family 2. Finally, in vitro functional analyses are needed to quantify the actual effect of the c.344G > A: p.(Arg115His) variant in the *LDLR* gene.

## Conclusions

The c.344G > A: p.(Arg115His) variant in the *LDLR* gene was not shared by the sibling with FH in Family 1, and patients with nonFH also had the variant in Family 2. In conclusion, the c.344G > A: p.(Arg115His) variant in the *LDLR* gene is interpreted as benign based on pedigree-based genetic analysis.

## Data Availability

The data are not available because some data are being used by another study.

## Abbreviations

APOB: Apolipoprotein B
ATT: Achilles tendon thickness
CAD: Coronary artery disease
FH: Familial hypercholesterolemia
HDL: High-density lipoprotein
HDL-C: HDL-cholesterol
LDL: Low-density lipoprotein
LDL-C: LDL-cholesterol
LDLR: LDL receptor
MLPA: Multiplex ligation-dependent probe amplification
PCSK9: Proprotein convertase subtilisin/kexin type 9
TC: Total cholesterol
TG: Triglycerides
